# Total retinal detachment in a morning glory disc anomaly

**DOI:** 10.11604/pamj.2022.43.24.33639

**Published:** 2022-09-16

**Authors:** Jihene Sayadi, Dhouha Gouider

**Affiliations:** 1 Department of Ophthalmology Hedi Rais Institute, Tunis El-Manar University, Tunis, Tunisia

**Keywords:** Morning glory disc anomaly, retinal detachment, optic disc abnormality

## Image in medicine

A six-year-old boy presented with poor vision and exotropia of his right eye (RE). His medical and ocular history was unremarkable. The visual acuity was limited to light perception in the RE. Slit-lamp examination of the anterior segment disclosed no abnormalities. Fundus examination of the RE revealed an enlarged funnel-shaped papillary excavation. The blood vessels appeared to increase in number, narrowed and radially arranged. Fundus examination also revealed a total retinal detachment (RD) with a proliferative vitreoretinopathy: retinal stiffness and folds with subretinal bands. There was no retinal tear. Examination of the left eye was unremarkable. We diagnosed a total RD associated with a morning glory disc (MGD) in the RE. Morning glory disc is a rare congenital condition. Authors reported RD in 37% of patients with MGD anomaly. The precise mechanism is still uncertain. Several etiologies have been suggested. The main ones were exudation from vessels, vitreous traction, retinal break on the optic disc, and migration of the cerebrospinal fluid from the subarachnoid space. There is no standard of care regarding RD with MGD anomaly.

**Figure 1:**
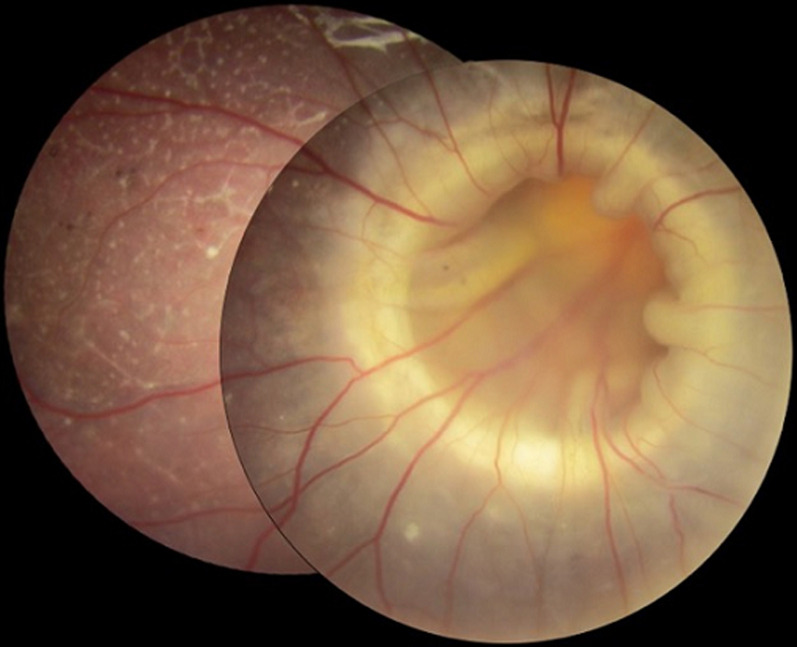
fundus photograph of the right eye showing retinal detachment associated with a morning glory disc anomaly

